# Assembly and analysis of the complete *Salix purpurea* L. (Salicaceae) mitochondrial genome sequence

**DOI:** 10.1186/s40064-016-3521-6

**Published:** 2016-10-28

**Authors:** Suyun Wei, Xuelin Wang, Changwei Bi, Yiqing Xu, Dongyang Wu, Ning Ye

**Affiliations:** 1 College of Forestry, Nanjing Forestry University, Nanjing, 210037 Jiangsu China; 2 The Southern Modern Forestry Collaborative Innovation Center, Nanjing Forestry University, Nanjing, 210037 Jiangsu China; 3 College of Information Science and Technology, Nanjing Forestry University, Nanjing, 210037 Jiangsu China; 4 School of Computer Science and Engineering, Southeast University, Nanjing, 211189 Jiangsu China

**Keywords:** Salicaceae, Mitochondrial genome, Genome assembly, Phylogenetic tree

## Abstract

Plant mitochondrial (mt) genomes possess several complex features, including a variable size, a dynamic genome structure, and complicated patterns of gene loss and gain throughout evolutionary history. Studies of plant mt genomes can, therefore, provide unique insights into organelle evolution. We assembled the complete *Salix purpurea* L. mt genome by screening genomic sequence reads generated by a Roche-454 pyrosequencing platform. The pseudo-molecule obtained has a typical circular structure 598,970 bp long, with an overall GC content of 55.06%. The *S. purpurea* mt genome contains 52 genes: 31 protein-coding, 18 tRNAs, and three rRNAs. Eighteen tandem repeats and 404 microsatellites are distributed unevenly throughout the *S. purpurea* mt genome. A phylogenetic tree of 23 representative terrestrial plants strongly supports *S. purpurea* inclusion in the Malpighiales clade. Our analysis contributes toward understanding the organization and evolution of organelle genomes in Salicaceae species.

**Electronic supplementary material:**

The online version of this article (doi:10.1186/s40064-016-3521-6) contains supplementary material, which is available to authorized users.

## Background

Mitochondria contribute to energy metabolism and play fundamental roles in plant development, fitness, and reproduction, as well as being associated with the biosynthesis of fatty acids and several active proteins (Mcbride et al. 2006; Ryan and Hoogenraad 2007). The mitochondrial (mt) genome has drawn increased attention during the genomic and now post-genomic eras owing to its maternal pattern of inheritance and unique evolutionary features, and is often used for the phylogenetic study of plants (Gualberto et al. 2014; Dames et al. 2015). Plant mt genomes can be extraordinarily larger than animal mt genomes, and vary significantly in size, even between very closely related species or within a single family (Alverson et al. 2010), whereas animal mt genomes, are conserved and relatively uniform in size (Zhang et al. 2012; Liu et al. 2013). More than 100 complete land plant mt genome sequences are available through the NCBI Organelle Genome Resources Web site (http://www.ncbi.nlm.nih.gov/genome/organelle/), ranging in size from 100,725 bp (*Buxbaumia aphylla*; GenBank accession number NC_024518) (Liu et al. 2014) to 1555.93 Kb (*Cucumis sativus*; GenBank accession number NC_016005) (Alverson et al. 2011b), since the first angiosperm mt genome nucleotide sequence was determined in 1997 (*Arabidopsis thaliana*; NC_001284) (Unseld et al. 1997). The comparative analysis of plant mt genomes enhances our understanding of genome rearrangement and DNA transfer mechanisms, and of phylogenetic diversity.

*Salix purpurea* L. is a willow species native to much of Europe (north to the British Isles, Poland, and the Baltic States), western Asia, and North Africa (Argus 1997; Skvortsov 1999; Sulima et al. 2009). It is a deciduous shrub growing 1–3 m tall, with purple-brown to yellow–brown shoots, green foliage, and small purple or red catkins produced in the early spring. *S. purpurea* has frequently been cultivated for its commercially important biomass. Purple willow bark contains a particularly valuable raw material traditionally used for the production of natural aspirin and other salicylic glycosides with analgesic, antipyretic, and anti-inflammatory effects (Skrzypczyńska 2001; Hakmaoui et al. 2007; Aliferis et al. 2015).

With the development of next generation sequencing (NGS) technologies, such as the Roche and Illumina platforms, new strategies are being used to characterize plant mitochondrial genomes. The mt genome of carrot (Zhang et al. 2012), soybean (Chang et al. 2013), rubber tree (Shearman et al. 2014), and some other species (Liu et al. 2013; Rd et al. 2015), have been successfully assembled through a combination approach using shotgun and paired-end NGS sequencing from non-enriched whole genome DNA libraries. Although the *S. purpurea* chloroplast genome has been published (Carlson et al. 2015), which is important for the genetic improvement and to further the understanding of biological mechanisms in plant species, the complete *S. purpurea* mt genome has not been previously published, because of its complex structure. In this study, we present the first complete mt genome of *S. purpurea*. We generated the mt genome sequence from 454 pyrosequencing whole genome big data. The mt genome was sequenced, assembled, and annotated as a circular-mapping DNA molecule. Additionally, we compared the *S. purpurea* mt genome to several previously published genomes to gain enhanced understanding of the evolution of organellar genomes. The strategy used in this study has broad applicability toward exploring additional mitochondrial genomes, and furthering the investigation of intra-cellular genome interactions and genome evolution.

## Methods

### Plant material

The raw sequencing and alignment data from the *S. purpurea* genome project is available at the NCBI Genome Resources Sequence Read Archive (SRA) database (http://www.ncbi.nlm.nih.gov/sra?LinkName=biosample_sra&from_uid=116760). The raw data were generated using Roche-454 FLX Titanium sequencing from random whole genome shotgun libraries. We deposited three whole genome sequence biosamples (Accessions: SRX029331, SRX029332, SRX029333), which respectively have 1,270,964 spots, 549,435 spots, and 448,379 spots, with total lengths of 1.4 Gb, 658.4 and 539 Mb.

### Genome assembly

Our research goal was to produce a gap-free, scaffold-level *S. purpurea* mt genome. Two random genomic 454 sequencing read samples were combined for assembly using the gsAssembler Java GUI in Newbler (version 2.7) with default parameters, producing 50, 115, 25, 100, and 17,094 assembled contigs from five separate runs. The initial contigs are a mixture of DNA from the nucleus and from organelles, therefore, BLASTN (Buhler et al. 2007) was used to isolate mitochondrial contigs from the whole genome reads based on plant mt genomes sequences downloaded from the NCBI Organelle Genome Resources. A total of 5831 contigs, with read depths between 50× and 100×, contained essential mitochondrial genes. We used Perl scripts to visualize contig connections from the Newbler assembly results, which records all contig read depth and connection information. False links to other contigs and a few wrong forks were removed manually, according to the read depth of the contigs. We connected 26 final contigs to produce a circular mt genome consistent with the standard structure of most mitochondrion genomes, and we mapped the sequence to the *Populus tremula* mt genome (NC_028096). The complete *S. purpurea* mt genome sequence is 598,970 bp long.

### Genome annotation

The *S. purpurea* mt genome was preliminarily annotated using the online program DOGMA (Organellar GenoMe Annotator) (Wyman et al. 2004) coupled with manual corrections for gene start and stop codons by comparison to homologous genes from other sequenced mt genomes. Subsequently, a detailed annotation of the protein-coding, rRNA, and tRNA genes was performed with a local database containing the nucleotide and protein sequences of all published land plant mitochondrial genomes available through the NCBI Organelle Genome Resources site. We also used tRNAscan-SE (Schattner et al. 2005) with default settings to corroborate tRNA boundaries identified by BLASTN. The circular mt genome map was drawn using Organellar Genome DRAW tool (OGDRAW) (Lohse et al. 2007) for further comparison of gene order and content.

### Repeat structure

Tandem repeats in the *S. purpurea* mt genome were identified using the Tandem Repeats Finder program (Benson 1999) with default settings. The Perl script MISA (Thiel et al. 2003) was used to detect simple sequence repeats (SSRs) with a motif size of one to six nucleotides and thresholds of eight, four, four, three, three, and three, respectively. All repeats identified by the various programs were manually confirmed to remove redundant results.

### Phylogenetic analysis

Phylogenetic analysis was performed with the mt genomes of 23 plant species, our newly sequenced *S. purpurea* mt genome and those from 22 other plant species (*Aegilops speltoides* [NC_022666], *Ajuga reptans* [NC_023103], *Batis maritima* [NC_024429], *Beta macrocarpa* [NC_015994], *Boea hygrometrica* [NC_016741], *Carica papaya* [NC_012116], *Citrullus lanatus* [NC_014043], *Cucumis sativus* [NC_016005], *Cucurbita pepo* [NC_014050], *Ginkgo biloba* [NC_027976], *Gossypium barbadense* [NC_028254], *Hyoscyamus niger* [NC_026515], *Liriodendron tulipifera* [NC_021152], *Phoenix dactylifera* [NC_016740], *Populus tremula* [NC_028096], *Salvia miltiorrhiza* [NC_023209], *Silene latifolia* [NC_014487], *Sorghum bicolor* [NC_008360], *Vitis vinifera* [NC_012119], *Zea luxurians* [NC_008333], *Zea mays subsp parviglumis* [NC_008332], and *Zea perennis* [NC_008331]).We obtained the 22 complete mt genome sequences through the NCBI Organelle Genome Resources Web site (http://www.ncbi.nlm.nih.gov/genome/organelle/). Twenty-three homologous protein-coding genes, 20 respiratory complex genes (*atp1*, *atp4*, *atp6*, *atp8*, *atp9*, *cob*, *cox1*, *cox2*, *cox3*, *nad1*, *nad2*, *nad3*, *nad4*, *nad4L*, *nad5*, *nad6*, *nad7*, *nad9*, *rps3*, and *rps4*), plus three cytochrome c biogenesis genes (*ccmB*, *ccmFc*, and *ccmFn*), were extracted from the 23 representative species mt genomes to estimate a phylogenetic tree. Exons of these genes were extracted and sequentially joined together using local Perl scripts. The orthologous genes were aligned using ClustalW (Thompson et al. 1994) and manually adjusted. A phylogenetic tree of the mitochondrial genome was estimated using the neighbor joining algorithm in MEGA version 6.0 (Tamura et al. 2013) with branch point confidence support based on 1000 bootstrap replicates.

## Results and discussion

### Genome features of the *S. purpurea*mitochondrial genome

We assembled the complete *S. purpurea*mt genome into a single circle of total length 598,970 bp from the *S. purpurea* whole genome project using Roche-454 Sequencing technologies. The sequence has been deposited in the NCBI GenBank Reference Sequence database with accession number NC_029693. We also deposited our *S. purpurea* mt genome data at GBROWSE (http://bio.njfu.edu.cn/gb2/gbrowse/Salix_pu_mt/). The overall GC content is 55.06%, with a base composition of 27.24% A, 27.82% T, 22.50% C, and 22.44% G (Table 1).

**Table 1:** Summary of the complete *S. purpurea* mitochondrial genome

Total mt genome size	598,970 bp
Number of unique genes	52
Number of protein coding genes	31
tRNA genes	18
rRNA genes	3
A content	27.24%
T content	27.82%
C content	22.50%
G content	22.44%
GC content	44.94%

**Fig. 1:**
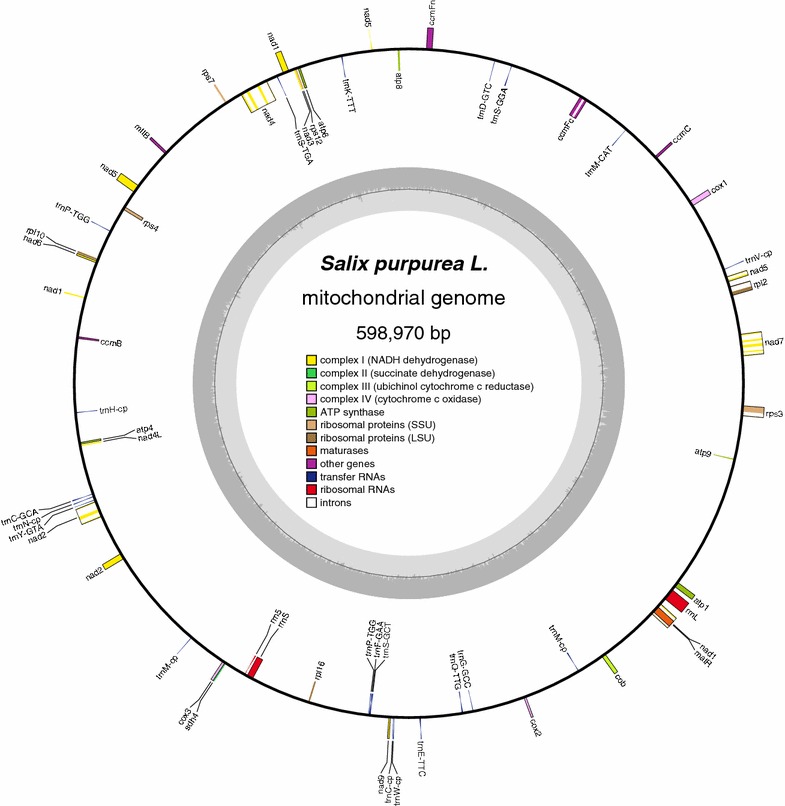
Gene map of the *S. purpurea* mitochondrial genome. Features on transcriptionally clockwise and counterclockwise strands are drawn on the inside and outside of the *outer circle*, respectively. Genes belonging to different functional groups are *color coded*. The innermost *darker gray shading* corresponds to GC content, while the *lighter gray* corresponds to AT content (colour figure online)

 The *S. purpurea* mt genome encodes 52 unique genes, consisting of three ribosomal RNA (rRNA; *rrn5*, *rrnL*, and *rrnS*) genes, 18 transfer RNA (tRNA) genes, and 31 protein-coding genes (PCGs) (Fig. 1). Among the 31 PCGs, nine code for subunits of NADH dehydrogenase (complex I; *nad1*, *nad2*, *nad3*, *nad4*, *nad4L*, *nad5*, *nad6*, *nad7*, and *nad9*), one for a subunit of succinate dehydrogenase (complex II; *sdh4*), one for a subunit of ubiquinol cytochrome c reductase (complex III; *cob*), three for subunits of cytochrome c oxidase (complexIV; *cox1*, *cox2*, and *cox3*), five for different subunits of ATP synthase (*atp1*, *atp4*, *atp6*, *atp8*, and *atp9*), four for small ribosomal subunits (SSU; *rps3*, *rps4*, *rps7*, and *rps12*), two for large ribosomal subunits (LSU; *rpl2* and *rpl16*), one for a maturase (*matR*), one for a SecY-independent transporter (*mttB*), and four are involved in the biogenesis of cytochrome c (*ccmB*, *ccmC*, *ccmFc*, and *ccmFn*). All 52 genes are single copy, with the exception of one tRNA gene (*trnP*-*UGG*), which has a duplicated copy, and one tRNA gene (*trnM*-*CAU*), which occurs in triplicate. Eight genes contain introns, with most being interrupted by a single or a pair of introns, except for *nad2* and *nad4*, which has three introns, and *nad7*, which has four introns (Table 2).

**Table 2:** List of genes identified in the *S. purpurea* mitochondrial genome

Group of gene	Name of gene		
Transfer RNAs	*trnC*-*GCA*	*trnC*-*ACA*	*trnD*-*GUC*
	*trnE*-*UUC*	*trnF*-*GAA*	*trnG*-*GCC*
	*trnH*-*GUG*	*trnK*-*UUU*	(×3)*trnM*-*CAU*
	*trnN*-*GUU*	(×2)*trnP*-*UGG*	*trnQ*-*UUG*
	*trnS*-*GGA*	*trnS*-*UGA*	*trnS*-*GCU*
	*trnV*-*GAC*	*trnW*-*CCA*	*trnY*-*GUA*
Ribosomal RNAs	*rrn5*	*rrnL*	*rrnS*
Complex I (NADH dehydrogenase)	*nad1* [2]	*nad2* [3]	*nad3*
	*nad4* [3]	*nad4L*	*nad5* [2]
	*nad6*	*nad7* [4]	*nad9*
Complex II (succinate dehydrogenase)	*sdh4*		
Complex III (ubichinol cytochrome c reductase)	*cob*		
Complex IV (cytochrome c oxidase)	*cox1*	*cox2*	*cox3*
ATP synthase	*atp1*	*atp4*	*atp6*
	*atp8*	*atp9*	
Ribosomal proteins (SSU)	*rps3* [1]	*rps4*	*rps7*
	*rps12*		
Ribosomal proteins (LSU)	*rpl2* [1]	*rpl16*	
Maturases	*matR*		
Other genes	*ccmB*	*ccmC*	*ccmFc* [1]
	*ccmFn*	*mttB*	

(×) number in parentheses indicates copy number of each gene

[] number in square brackets indicates intron number of each gene

The positions of all the genes identified in the *S. purpurea* mt genome and profiles of those genes are presented in Table 3. Protein-coding genes range in length from 2004 bp (*nad5*) to 225 bp (*atp9*). Most of the PCGs use ATG as the start codon, except for *mttB*, which starts with ATT, and *rpl16*, which starts with GTG. Fifteen PCGs (*rpl2*, *cox1*, *atp6*, *nad3*, *rps7*, *nad5*, *rps4*, *nad1*, *nad4L*, *nad2*, *sdh4*, *rpl16*, *nad9*, *cox2*, and *rps3*) use the stop codon TAA; eight PCGs (*nad7*, *ccmFc*, *atp8*, *mttB*, *nad6*, *atp4*, *matR*, and *atp9*) use the stop codon TAG, and eight PCGs (*ccmC*, *ccmFn*, *rps12*, *nad4*, *ccmB*, *cox3*, *cob*, and *atp1*) use the stop codon TGA.

**Table 3:** Gene profile and organization of the *S. purpurea* mitogenome

Gene	Position	Size (bp)	Start codon	Stop codon
*nad7*	7801–13973	1185	ATG	TAG
*rpl2*	25266–27874	1032	ATG	TAA
*tRNA* ^*Val*^-*GAC*	33191–33262	72	–	–
*cox1*	53262–54845	1584	ATG	TAA
*ccmC*	70277–71029	753	ATG	TGA
*tRNA* ^*Met*^-*CAU*	82261–82334	74	–	–
*ccmFc*	96139–98429	1356	ATG	TAG
*tRNA* ^*Ser*^-*GGA*	119917–120002	86	–	–
*tRNA* ^*Asp*^-*GUC*	124892–124965	74	–	–
*ccmFn*	143062–144786	1725	ATG	TGA
*atp8*	152417–152890	474	ATG	TAG
*tRNA* ^*Lys*^-*UUU*	169117–169189	73	–	–
*atp6*	181320–182033	714	ATG	TAA
*rps12*	182412–182789	378	ATG	TGA
*nad3*	182834–183190	357	ATG	TAA
*tRNA* ^*Ser*^-*UGA*	188249–188335	87	–	–
*nad4*	191566–199965	1488	ATG	TGA
*rps7*	204526–204972	447	ATG	TAA
*mttB*	226705–227490	786	ATT	TAG
*nad5*	29347–241532	2004	ATG	TAA
*rps4*	246410–247378	969	ATG	TAA
*tRNA* ^*Pro*^-*UGG*	254501–254575	75	–	–
*nad6*	263829–264458	630	ATG	TAG
*nad1*	186306–530338	888	ATG	TAA
*ccmB*	286077–286691	615	ATG	TGA
*tRNA* ^*His*^-*GUG*	307813–307886	74	–	–
*atp4*	316441–317037	597	ATG	TAG
*nad4L*	317272–317574	303	ATG	TAA
*tRNA* ^*Cys*^-*GCA*	331569–331639	71	–	–
*tRNA* ^*Asn*^-*GUU*	332440–332511	72	–	–
*tRNA* ^*Tyr*^-*GUA*	333372–333454	83	–	–
*nad2*	350407–352128	1461	ATG	TAA
*tRNA* ^*Met*^-*CAU*	381780–381852	73	–	–
*cox3*	393013–393810	798	ATG	TGA
*sdh4*	393738–394133	396	ATG	TAA
*rrn5*	400296–400410	115	–	–
*rrnS*	401034–402945	1912	–	–
*rpl16*	420055–420465	411	GTG	TAA
*tRNA* ^*Pro*^-*UGG*	437597–437671	75	–	–
*tRNA* ^*Phe*^-*GAA*	437924–437997	74	–	–
*tRNA* ^*Ser*^-*GCU*	438171–438258	88	–	–
*nad9*	443456–444028	573	ATG	TAA
*tRNA* ^*Cys*^-*ACA*	444810–444880	71	–	–
*tRNA* ^*Trp*^-*CCA*	445041–445114	74	–	–
*tRNA* ^*Glu*^-*UUC*	452452–452523	72	–	–
*tRNA* ^*Glu*^-*UUG*	464581–464652	72	–	–
*tRNA* ^*Gly*^-*GCC*	467557–467628	72	–	–
*cox2*	482620–483294	675	ATG	TAA
*tRNA* ^*Met*^-*CAU*	499876–499948	73	–	–
*cob*	507832–509013	1182	ATG	TGA
*matR*	527282–529225	1944	ATG	TAG
*rrnL*	532221–535541	3321	–	–
*atp1*	536676–538199	1524	ATG	TGA
*atp9*	576927–577151	225	ATG	TAG
*rps3*	589666–592572	1644	ATG	TAA

### Analysis of tandem repeats and SSRs

Tandem repeats (TRs) are DNA sequence motifs that play an important role in genome recombination and rearrangement (Cavalier-Smith 2002; Zhao et al. 2013), and are often used for population and phylogenetic analyses (Nie et al. 2012; Schaper and Anisimova 2015). We found 18 tandem repeats in the *S. purpurea* mt genome with lengths ranging from 4 to 28 bp (Table 4). Most of the repeats (94%) were distributed in non-coding regions, specifically: 83% in intergenic spacer regions, 11% in introns, and 6% in protein-coding regions.

**Table 4:** Tandem repeat sequences in the *S. purpurea* mt genome

No.	Size (bp)	Location	Repeat
1	14	IGS(*nad5*, *trnV*-*GAC*)	TTTAAGAATACCGA (×2)
2	13	IGS(*trnV*-*GAC,cox1*)	TTAGTTTATGAAT (×2)
3	15	IGS(*trnM*-*CAT*, *ccmFc*)	ATTATAGGATTATATT (×2.1)
4	21	IGS(*ccmFc*, *trnS*-*GGA*)	TATTATAAGATCATCTCACCT (×2)
5	19	IGS(*ccmFc*, *trnS*-*GGA*)	TTTTCTTCTTGCTTCTGTT (×2.1)
6	20	IGS(*atp8*, *nad5*)	AGAGTATGAAAGAACAGAAT (×2)
7	13	IGS(*atp8*, *nad5*)	AAGAATGAATTAC (×2.2)
8	15	*nad1*	TAAAAAAAAAAAGGC (×2)
9	28	IGS(*rps4*, *trnP*-*TGG*)	TATAAAGAAAGACCTTGTACATCTGTCC (×2.1)
10	22	IGS(*trnP*-*TGG*, *rpl10*)	TTTCTTCCCTCTCTATAGCCTA (×2)
11	4	IGS*(atp1,trnH*-*GTG)*	CTTT (×6.5)
12	25	IGS*(atp1,trnH*-*GTG)*	TCGACTGTTAAGGACACAGAGGGGA (×1.9)
13	22	IGS*(atp1,trnH*-*GTG)*	TTCGTGTACCAATTTCAGTGGT (×2)
14	14	IGS(*trnN*-*GTT*, *trnY*-*GTA*)	TTAGGTAGGATAGA (×2.1)
15	7	*nad2 (intron)*	CTTATAT (×4)
16	18	*nad2 (intron)*	AACATTATAAGAAAAGAT(×2.1)
17	24	IGS(*rpl16,trnP*-*TGG*)	CATAACCAGGCAGTGAGGAATCTT (×2)
18	13	IGS(*trnG*-*GCC,cox2*)	AATAAGAATAATA (×2.8)

**Fig. 2:**
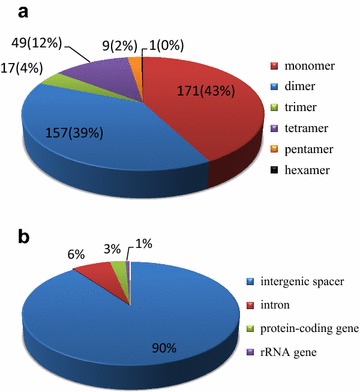
Total SSR distribution in the *S. purpurea* mitochondrial genome. **a** SSR distribution according to type: mononucleotide, dinucleotide, trinucleotide, pentanucleotide, and hexanucleotide repeats. SSR number and percentage (in *brackets*) are provided. **b** SSR distribution among four different regions: intergenic spacer, intron, protein-coding, and rRNA

Simple sequence repeats (SSRs), also known as microsatellites, are short tandem repeat sequences with repeat lengths generally between one and six base pairs per unit, and are extensively distributed throughout mitochondrial genomes (Provan et al. 2001; Chen et al. 2006). SSRs are important genetic molecular markers, widely used in assisted breeding (Rafalski and Tingey 1993), population genetics (Doorduin et al. 2011; He et al. 2012; Powell et al. 1995), plant typing (Xue et al. 2012; Yang et al. 2011), and genetic linkage map construction (Pugh et al. 2004). We identified 404 SSR motifs in the *S. purpurea* mt genome with the microsatellite identification tool MISA (Thiel et al. 2003), accounting for 3810 bp of the total sequence. Among these SSRs, 171 have mononucleotide, 157 have dinucleotide, 17 have trinucleotide, 49 have tetranucleotide, nine have pentanucleotide, and one has hexanucleotide repeat motifs (Fig. 2a). Most of the mononucleotide repeats (90.7%) are composed of A/T, the 23 dinucleotides are all composed entirely of AT/TA, and the rest of the SSRs also have a high A/T content (Additional file 1: Table S1). These results are consistent with observations that mitochondrial SSRs are generally composed of short polyadenine (polyA) or polythymidine (polyT) repeats (Kuang et al. 2011). The high A/T content in mitochondrial SSRs contributes to a biased composition, such that the overall AT content is 55.06% in the *S. purpurea* mt genome. Moreover, it is clear that SSRs are most abundant in intergenic spacers versus other regions, and these account for 90.35% of all SSRs detected. The remaining 6.44, 2.48, and 0.74% of SSRs are in introns, protein-coding regions, and rRNA regions, respectively (Fig. 2b).

### Comparison with other mitochondrial genomes

Multiple complete mt genomes provide an opportunity to compare variation in size, structure, and sequence content at the genomic level (Alverson et al. 2011a). We selected 35 land plant mt genomes and compared features to observe the variation among them and the *S. purpurea* mt genome (Additional file 1: Table S2). The mt genome size of our samples ranges from 104,239 bp in *Anomodon rugelii* to 982,833 bp in *Cucurbita pepo*, and the GC content ranges from 39.93% in *Bucklandiella orthotrichacea* to 53.02% in *Welwitschia mirabilis*. Because of a large number of open reading frames (ORFs) coding for proteins of unknown function in plant mt genomes, and frequent plastid DNA insertions including mitochondrial tRNA genes (Notsu et al. 2002; Marechal-Drouard et al. 1990), the number of genes in plant mt genomes widely vary. Some examples include 12 protein-coding genes in *Viscum album* versus 193 in *Capsicum annuum*, six tRNAs *Viscum album* versus 34 in *Phlegmariurus squarrosus*, and one rRNA *Viscum album* versus nine in *Triticum aestivum*.

We particularly compared the *S. purpurea* mt genome with the *Populus tremula* mt genome (NC_028096), another member of the Salicaceae family. The *P. tremula* mt genome is 783,442 bp long, which is much larger than that of *S. purpurea*, however, its base composition of 27.62% A, 22.36% C, 22.38% G, 27.64% T, with a slight A + T bias of 55.25%, is similar to that of the *S. purpurea* mt genome. As described previously, the complete *P. tremula* mt genomehas three rRNA genes, 22 tRNA genes, and 33 protein-coding genes. Upon a comparison of all orthologous genes between the two genomes, three PCGs (*rpl10, rps1,* and *rps14*) and three tRNA genes (*trnH*-*AUG*, *trnK*-*CUU*, and *trnS*-*UGU*) are seen to be present in the *P. tremula* genome, but not in the *S. purpurea* genome, while only two tRNA genes (*trnC*-*ACA* and *trnV*-*GAC*) exist in the *S. purpurea* genome that do not exist in the *P. tremula* genome. The *P. tremula* genome has 838 bp of tandem repeats, while *S. purpurea* has 665 bp (Table 5). The *S. purpurea* mitogenome, with its smaller gene count, sparser PCG annotation, and fewer tandem repeat, compared with *P. tremula*, may provide insight to further understand the divergent evolution between willow and poplar.

**Table 5:** Summary of the complete *Populus tremula* mitochondrial genome

Total mt genome size	783,442 bp
Number of genes	59
Protein-coding genes	33
tRNA genes	22
rRNA genes	3
A content	27.62%
T content	27.64%
C content	22.36%
G content	22.38%
GC content	44.75%
Total tandom repeats size	838 bp

### Phylogenetic analysis

**Fig. 3:**
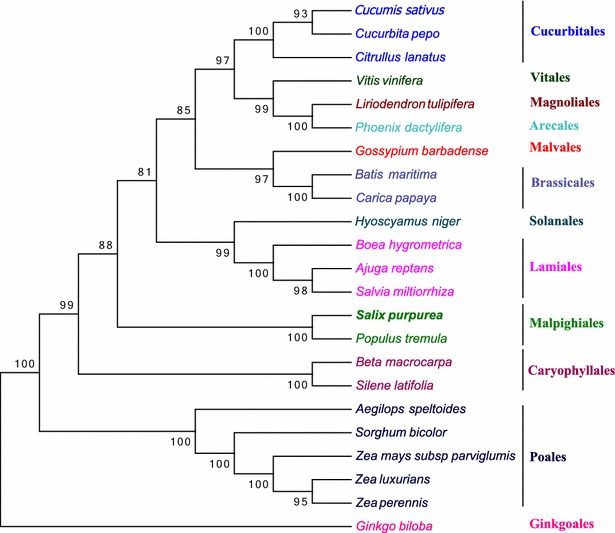
Phylogenetic tree of representative higher plant mitochondrial genomes. The phylogenetic tree was constructed using the neighbor joining method with 23 mitochondrial protein-coding genes from 23 representative plant mitochondrial genomes. Numbers at the nodes are bootstrap support values. *G. biloba* was designated the outgroup. Taxonomic orders were indicated at right

The dramatic increase in the number of sequenced mt genomes provided by NGS technology can yield unique insights into the phylogenetic relationships among plants. We estimated a plant phylogeny based on 23 conserved, orthologous mt genes (*atp1*, *atp4*, *atp6*, *atp8*, *atp9*, *cob*, *cox1*, *cox2*, *cox3*, *nad1*, *nad2*, *nad3*, *nad4*, *nad4L*, *nad5*, *nad6*, *nad7*, *nad9*, *rps3*, *rps4*, *ccmB*, *ccmFc*, and *ccmFn*) from 23 representative higher plant species (*Cucumis sativus*, *Cucurbita pepo*, *Citrullus lanatus*, *Vitis vinifera*, *Liriodendron tulipifera*, *Phoenix dactylifera*, *Gossypium barbadense*, *Batis maritima*, *Carica papaya*, *Hyoscyamus niger*, *Boea hygrometrica*, *Ajuga reptans*, *Salvia miltiorrhiza*, *Salix purpurea*, *Populus tremula*, *Beta macrocarpa*, *Silene latifolia*, *Aegilops speltoides*, *Sorghum bicolor*, *Zea mays subsp parviglumis*, *Zea luxurians*, *Zea perennis,* and *Ginkgo biloba*). Among these species, 22 are angiosperms representing 11 orders: Arecales (*Phoenix dactylifera*), Brassicales (*Batis maritima* and *Carica papaya*), Caryophyllales (*Beta macrocarpa* and *Silene latifolia*), Cucurbitales (*Citrullus lanatus*, *Cucumis sativus*, and *Cucurbita pepo*), Lamiales (*Ajuga reptans*, *Boea hygrometrica*, and *Salvia miltiorrhiza*), Magnoliales (*Liriodendron tulipifera*), Malpighiales (*Populus tremula* and *Salix purpurea*), Malvales (*Gossypium barbadense*), Poales (*Aegilops speltoides, Sorghum bicolor, Zea luxurians, Zea mays subsp parviglumis,* and *Zea perennis*), Solanales (*Hyoscyamus niger*), and Vitales (*Vitis vinifera*) (Additional file 1: Table S3). One additional species, a gymnosperm, *Ginkgo biloba*, was designated the outgroup. We estimated a phylogenetic tree of these species using the neighbor-joining method (NJ; Fig. 3). Bootstrap analysis shows 20 of 23 nodes with bootstrap values > 90%, and 18 of these have a bootstrap value of 100%. Our phylogenetic analysis strongly supports the close relationship of *S. purpurea* and *P. tremula*, with a 100% bootstrap value. Both are classified as members of the Salicaceae family, and our results are consistent with previous molecular and taxonomic studies.

## Conclusions

The mitochondrial genome is proving to be an effective and important tool for gaining insight into species evolution. Plant mt genomes have striking differences in structure, size, gene order, and gene content. This has generated significant interest in exploring and further understanding plant mitochondrion evolution. Our investigation of the complete *S. purpurea* mt genome is an important addition to the limited amount of genomic data available for the Salicaceae. The *S. purpurea* mt genome possesses most of the common characteristics of higher plant mt genomes. Our comparative and phylogenetic analyses should contribute to a more comprehensive understanding of mitochondrion molecular evolution in higher plants.

## Additional file

**Additional file 1: Table S1.** Distribution of SSRs in the S. purpurea mitochondrial genome. **Table S2** Comparison of basic features among 35 mitochondrial genomes. **Table S3** The list of mitochondrial genome sequences used in phylogenetic study.
