# Supplementary material for: Assembly and analysis of the complete Salix purpurea L. (Salicaceae) mitochondrial genome sequence
Source: Springerplus. 2016 Oct 28;5(1):1894. doi: 10.1186/s40064-016-3521-6 (PMC5084139; doi:10.1186/s40064-016-3521-6)
Supplement: Supplementary file 1 — Additional file 1: Table S1. Distribution of SSRs in the S. purpurea mitochondrial genome. Table S2 Comparison of basic features among 35 mitochondrial genomes. Table S3 The list of mitochondrial genome sequences used in phylogenetic study. [file 40064_2016_3521_MOESM1_ESM.docx]

**Table S1.** Distribution of SSRs in the *S. purpurea* mitochondrial genome.

| **Type** | **SSR** | **No. of Repeats** | **Start** | **End** | **Location** |
| --- | --- | --- | --- | --- | --- |
| **monomer** | (A)9 | 9 | 2973 | 2981 | IGS (*rps3, nad7*) |
|  | (A)8 | 8 | 5745 | 5752 | IGS (*rps3, nad7*) |
|  | (T)10 | 10 | 6758 | 6767 | IGS (*rps3, nad7*) |
|  | (T)8 | 8 | 7393 | 7400 | IGS (*rps3, nad7*) |
|  | (T)9 | 9 | 7657 | 7665 | IGS (*rps3, nad7*) |
|  | (A)8 | 8 | 13322 | 13329 | *nad7* (intron) |
|  | (A)11 | 11 | 14022 | 14032 | IGS (*nad7, rpl2*) |
|  | (T)10 | 10 | 28446 | 28455 | IGS (*rpl2, nad5*) |
|  | (T)8 | 8 | 41483 | 41490 | IGS (*trnV-GAC, cox1*) |
|  | (T)8 | 8 | 42108 | 42115 | IGS (*trnV-GAC, cox1*) |
|  | (T)11 | 11 | 43886 | 43896 | IGS (*trnV-GAC, cox1*) |
|  | (A)9 | 9 | 44359 | 44367 | IGS (*trnV-GAC, cox1*) |
|  | (T)11 | 11 | 44651 | 44661 | IGS (*trnV-GAC, cox1*) |
|  | (A)8 | 8 | 46405 | 46412 | IGS (*trnV-GAC, cox1*) |
|  | (T)8 | 8 | 64731 | 64738 | IGS (*cox1, ccmC*) |
|  | (T)8 | 8 | 66581 | 66588 | IGS (*cox1, ccmC*) |
|  | (T)8 | 8 | 72421 | 72428 | IGS (*ccmC, trnM-CAT*) |
|  | (T)9 | 9 | 78646 | 78654 | IGS (*ccmC, trnM-CAT*) |
|  | (T)8 | 8 | 80407 | 80414 | IGS (*ccmC, trnM-CAT*) |
|  | (A)10 | 10 | 82723 | 82732 | IGS (*trnM-CAT,ccmFc*) |
|  | (C)9 | 9 | 88601 | 88609 | IGS (*trnM-CAT,ccmFc*) |
|  | (T)9 | 9 | 89108 | 89116 | IGS (*trnM-CAT,ccmFc*) |
|  | (A)8 | 8 | 93354 | 93361 | IGS (*trnM-CAT,ccmFc*) |
|  | (A)8 | 8 | 96265 | 96272 | *ccmFc* |
|  | (A)8 | 8 | 96900 | 96907 | *ccmFc* (intron) |
|  | (C)8 | 8 | 97811 | 97818 | *ccmFc* |
|  | (A)11 | 11 | 99660 | 99670 | IGS (*ccmFc,trnS-GCA*) |
|  | (A)8 | 8 | 102507 | 102514 | IGS (*ccmFc,trnS-GCA*) |
|  | (T)9 | 9 | 104548 | 104556 | IGS (*ccmFc,trnS-GCA*) |
|  | (A)8 | 8 | 107352 | 107359 | IGS (*ccmFc,trnS-GCA*) |
|  | (A)9 | 9 | 119482 | 119490 | IGS (*ccmFc,trnS-GCA*) |
|  | (A)9 | 9 | 120111 | 120119 | IGS (*trnS-GCA,trnD-GTC*) |
|  | (T)10 | 10 | 120362 | 120371 | IGS (*trnS-GCA,trnD-GTC*) |
|  | (A)8 | 8 | 121161 | 121168 | IGS (*trnS-GCA,trnD-GTC*) |
|  | (C)8 | 8 | 122197 | 122204 | IGS (*trnS-GCA,trnD-GTC*) |
|  | (A)9 | 9 | 124692 | 124700 | IGS (*trnS-GCA,trnD-GTC*) |
|  | (A)9 | 9 | 125068 | 125076 | IGS (*trnD-GTC,ccmFn*) |
|  | (A)10 | 10 | 126962 | 126971 | IGS (*trnD-GTC,ccmFn*) |
|  | (T)9 | 9 | 128852 | 128860 | IGS (*trnD-GTC,ccmFn*) |
|  | (A)11 | 11 | 130549 | 130559 | IGS (*trnD-GTC,ccmFn*) |
|  | (T)8 | 8 | 131507 | 131514 | IGS (*trnD-GTC,ccmFn*) |
|  | (A)9 | 9 | 132159 | 132167 | IGS (*trnD-GTC,ccmFn*) |
|  | (A)11 | 11 | 132984 | 132994 | IGS (*trnD-GTC,ccmFn*) |
|  | (A)8 | 8 | 138321 | 138328 | IGS (*trnD-GTC,ccmFn*) |
|  | (A)11 | 11 | 140966 | 140976 | IGS (*trnD-GTC,ccmFn*) |
|  | (A)10 | 10 | 145541 | 145550 | IGS (*ccmFn,atp8*) |
|  | (T)8 | 8 | 149362 | 149369 | IGS (*ccmFn,atp8*) |
|  | (T)8 | 8 | 152596 | 152603 | *atp8* |
|  | (T)9 | 9 | 159790 | 159798 | IGS (*atp8,nad5*) |
|  | (A)8 | 8 | 163857 | 163864 | IGS (*nad5,trnK-TTT*) |
|  | (A)9 | 9 | 171103 | 171111 | IGS (*trnK-TTT,atp6*) |
|  | (A)11 | 11 | 175648 | 175658 | IGS (*trnK-TTT,atp6*) |
|  | (A)8 | 8 | 179143 | 179150 | IGS (*trnK-TTT,atp6*) |
|  | (A)8 | 8 | 183241 | 183248 | IGS (*nad3,nad1*) |
|  | (T)8 | 8 | 184092 | 184099 | IGS (*nad3,nad1*) |
|  | (A)11 | 11 | 185559 | 185569 | *nad1* (intron) |
|  | (A)11 | 11 | 189150 | 189160 | IGS (*trnS-TGA,nad4*) |
|  | (A)8 | 8 | 190704 | 190711 | IGS (*trnS-TGA,nad4*) |
|  | (T)10 | 10 | 192165 | 192174 | *nad4* (intron) |
|  | (C)8 | 8 | 194792 | 194799 | *nad4* (intron) |
|  | (C)9 | 9 | 195087 | 195095 | *nad4* (intron) |
|  | (T)8 | 8 | 201670 | 201677 | IGS (*nad4,rps7*) |
|  | (T)8 | 8 | 212300 | 212307 | IGS (*rps7,mttB*) |
|  | (C)8 | 8 | 214846 | 214853 | IGS (*rps7,mttB*) |
|  | (A)10 | 10 | 227853 | 227862 | IGS (*mttB,nad5*) |
|  | (A)8 | 8 | 228821 | 228828 | IGS (*mttB,nad5*) |
|  | (T)8 | 8 | 249928 | 249935 | IGS (*rps4,trnP-TGG*) |
|  | (T)9 | 9 | 250378 | 250386 | IGS (*rps4,trnP-TGG*) |
|  | (A)8 | 8 | 251559 | 251566 | IGS (*rps4,trnP-TGG*) |
|  | (T)9 | 9 | 252702 | 252710 | IGS (*rps4,trnP-TGG*) |
|  | (T)10 | 10 | 253679 | 253688 | IGS (*rps4,trnP-TGG*) |
|  | (T)9 | 9 | 256320 | 256328 | IGS (*trnP-TGG,rpl10*) |
|  | (T)8 | 8 | 257793 | 257800 | IGS (*trnP-TGG,rpl10*) |
|  | (A)10 | 10 | 259308 | 259317 | IGS (*trnP-TGG,rpl10*) |
|  | (T)8 | 8 | 261271 | 261278 | IGS (*trnP-TGG,rpl10*) |
|  | (T)9 | 9 | 267538 | 267546 | IGS (*nad6,nad1*) |
|  | (A)9 | 9 | 268121 | 268129 | IGS (*nad6,nad1*) |
|  | (A)10 | 10 | 276712 | 276721 | IGS (*nad1,ccmB*) |
|  | (T)8 | 8 | 281117 | 281124 | IGS (*nad1,ccmB*) |
|  | (C)9 | 9 | 286980 | 286988 | IGS (*ccmB,atp1*) |
|  | (T)8 | 8 | 299998 | 300005 | IGS (*atp1,trnH-GTG*) |
|  | (T)8 | 8 | 305413 | 305420 | IGS (*atp1,trnH-GTG*) |
|  | (T)8 | 8 | 308875 | 308882 | IGS (*trnH-GTG,atp4*) |
|  | (A)10 | 10 | 309160 | 309169 | IGS (*trnH-GTG,atp4*) |
|  | (T)9 | 9 | 310347 | 310355 | IGS (*trnH-GTG,atp4*) |
|  | (A)8 | 8 | 314202 | 314209 | IGS (*trnH-GTG,atp4*) |
|  | (A)10 | 10 | 326545 | 326554 | IGS (*nad4L,trnC-GCA*) |
|  | (T)8 | 8 | 326714 | 326721 | IGS (*nad4L,trnC-GCA*) |
|  | (T)8 | 8 | 327287 | 327294 | IGS (*nad4L,trnC-GCA*) |
|  | (A)9 | 9 | 330627 | 330635 | IGS (*nad4L,trnC-GCA*) |
|  | (A)9 | 9 | 332368 | 332376 | IGS (*nad4L,trnC-GCA*) |
|  | (A)9 | 9 | 334561 | 334569 | *nad2* (intron) |
|  | (A)10 | 10 | 335257 | 335266 | *nad2* (intron) |
|  | (T)9 | 9 | 337350 | 337358 | *nad2* (intron) |
|  | (C)9 | 9 | 337713 | 337721 | *nad2* (intron) |
|  | (A)8 | 8 | 348157 | 348164 | *nad2* (intron) |
|  | (T)9 | 9 | 352962 | 352970 | IGS (*nad2,trnM-CAT*) |
|  | (A)8 | 8 | 353990 | 353997 | IGS (*nad2,trnM-CAT*) |
|  | (T)9 | 9 | 354169 | 354177 | IGS (*nad2,trnM-CAT*) |
|  | (A)11 | 11 | 356567 | 356577 | IGS (*nad2,trnM-CAT*) |
|  | (T)9 | 9 | 359348 | 359356 | IGS (*nad2,trnM-CAT*) |
|  | (T)10 | 10 | 374524 | 374533 | IGS (*nad2,trnM-CAT*) |
|  | (A)10 | 10 | 382746 | 382755 | IGS (*trnM-CAT,cox3*) |
|  | (A)10 | 10 | 383473 | 383482 | IGS (*trnM-CAT,cox3*) |
|  | (T)9 | 9 | 389017 | 389025 | IGS (*trnM-CAT,cox3*) |
|  | (T)8 | 8 | 393309 | 393316 | *cox3* |
|  | (A)9 | 9 | 395014 | 395022 | IGS (*sdh4,rrn5*) |
|  | (A)9 | 9 | 395773 | 395781 | IGS (*sdh4,rrn5*) |
|  | (A)9 | 9 | 397225 | 397233 | IGS (*sdh4,rrn5*) |
|  | (C)8 | 8 | 402411 | 402418 | *rrnS* |
|  | (T)9 | 9 | 404553 | 404561 | IGS (*rrnS,rpl16*) |
|  | (A)9 | 9 | 407765 | 407773 | IGS (*rrnS,rpl16*) |
|  | (T)9 | 9 | 417578 | 417586 | IGS (*rrnS,rpl16*) |
|  | (T)10 | 10 | 419864 | 419873 | IGS (*rrnS,rpl16*) |
|  | (T)9 | 9 | 427517 | 427525 | IGS (*rpl16,trnP-TGG*) |
|  | (T)9 | 9 | 430308 | 430316 | IGS (*rpl16,trnP-TGG*) |
|  | (A)10 | 10 | 438070 | 438079 | IGS (*trnF-GAA,trnS-GCT*) |
|  | (T)13 | 13 | 439609 | 439621 | IGS (*trnS-GCT,nad9*) |
|  | (T)9 | 9 | 444255 | 444263 | IGS (*nad9,trnC-ACA*) |
|  | (C)11 | 11 | 446690 | 446700 | IGS (*trnW-CCA,trnE-TTC*) |
|  | (T)11 | 11 | 452181 | 452191 | IGS (*trnW-CCA,trnE-TTC*) |
|  | (T)10 | 10 | 452891 | 452900 | IGS (*trnE-TTC,trnQ-TTG*) |
|  | (T)10 | 10 | 453924 | 453933 | IGS (*trnE-TTC,trnQ-TTG*) |
|  | (T)9 | 9 | 458169 | 458177 | IGS (*trnE-TTC,trnQ-TTG*) |
|  | (C)8 | 8 | 459190 | 459197 | IGS (*trnE-TTC,trnQ-TTG*) |
|  | (T)8 | 8 | 463130 | 463137 | IGS (*trnE-TTC,trnQ-TTG*) |
|  | (T)9 | 9 | 467780 | 467788 | IGS (*trnG-GCC,cox2*) |
|  | (T)10 | 10 | 468283 | 468292 | IGS (*trnG-GCC,cox2*) |
|  | (T)8 | 8 | 472544 | 472551 | IGS (*trnG-GCC,cox2*) |
|  | (T)10 | 10 | 482041 | 482050 | IGS (*trnG-GCC,cox2*) |
|  | (T)9 | 9 | 483893 | 483901 | IGS (*cox2,trnM-CAT*) |
|  | (A)8 | 8 | 487302 | 487309 | IGS (*cox2,trnM-CAT*) |
|  | (A)8 | 8 | 491137 | 491144 | IGS (*cox2,trnM-CAT*) |
|  | (T)11 | 11 | 495694 | 495704 | IGS (*cox2,trnM-CAT*) |
|  | (T)10 | 10 | 496983 | 496992 | IGS (*cox2,trnM-CAT*) |
|  | (T)9 | 9 | 498409 | 498417 | IGS (*cox2,trnM-CAT*) |
|  | (T)8 | 8 | 499153 | 499160 | IGS (*cox2,trnM-CAT*) |
|  | (G)8 | 8 | 500033 | 500040 | IGS (*trnM-CAT,cob*) |
|  | (A)10 | 10 | 500684 | 500693 | IGS (*trnM-CAT,cob*) |
|  | (A)9 | 9 | 501681 | 501689 | IGS (*trnM-CAT,cob*) |
|  | (T)8 | 8 | 501991 | 501998 | IGS (*trnM-CAT,cob*) |
|  | (A)9 | 9 | 505426 | 505434 | IGS (*trnM-CAT,cob*) |
|  | (T)8 | 8 | 505782 | 505789 | IGS (*trnM-CAT,cob*) |
|  | (A)10 | 10 | 515372 | 515381 | IGS (*cob,nad1*) |
|  | (A)9 | 9 | 523269 | 523277 | IGS (*cob,nad1*) |
|  | (A)8 | 8 | 525745 | 525752 | IGS (*cob,nad1*) |
|  | (T)10 | 10 | 526612 | 526621 | IGS (*cob,nad1*) |
|  | (G)8 | 8 | 526960 | 526967 | *nad1* (intron) |
|  | (A)9 | 9 | 532126 | 532134 | IGS (*nad1,rrnL*) |
|  | (G)8 | 8 | 534888 | 534895 | *rrnL* |
|  | (A)8 | 8 | 538377 | 538384 | IGS (*atp1,atp9*) |
|  | (T)10 | 10 | 542904 | 542913 | IGS (*atp1,atp9*) |
|  | (A)8 | 8 | 554335 | 554342 | IGS (*atp1,atp9*) |
|  | (A)10 | 10 | 560107 | 560116 | IGS (*atp1,atp9*) |
|  | (T)9 | 9 | 562192 | 562200 | IGS (*atp1,atp9*) |
|  | (T)8 | 8 | 565082 | 565089 | IGS (*atp1,atp9*) |
|  | (T)10 | 10 | 568379 | 568388 | IGS (*atp1,atp9*) |
|  | (T)8 | 8 | 571495 | 571502 | IGS (*atp1,atp9*) |
|  | (A)9 | 9 | 572423 | 572431 | IGS (*atp1,atp9*) |
|  | (A)8 | 8 | 572813 | 572820 | IGS (*atp1,atp9*) |
|  | (C)9 | 9 | 574307 | 574315 | IGS (*atp1,atp9*) |
|  | (T)10 | 10 | 577811 | 577820 | IGS (*atp9,rps3*) |
|  | (T)9 | 9 | 584322 | 584330 | IGS (*atp9,rps3*) |
|  | (T)10 | 10 | 587661 | 587670 | IGS (*atp9,rps3*) |
|  | (T)9 | 9 | 590354 | 590362 | *rps3* (intron) |
|  | (T)8 | 8 | 591968 | 591975 | *rps3* |
|  | (A)8 | 8 | 592555 | 592562 | *rps3* |
|  | (A)8 | 8 | 592951 | 592958 | IGS (*rps3, nad7*) |
|  | (T)8 | 8 | 594635 | 594642 | IGS (*rps3, nad7*) |
|  | (A)9 | 9 | 597348 | 597356 | IGS (*rps3, nad7*) |
|  | (C)8 | 8 | 598963 | 598970 | IGS (*rps3, nad7*) |
| **dimer** | (TC)4 | 8 | 2119 | 2126 | IGS (*rps3, nad7*) |
|  | (TG)4 | 8 | 2603 | 2610 | IGS (*rps3, nad7*) |
|  | (TA)4 | 8 | 8609 | 8616 | *nad7* (intron) |
|  | (GA)4 | 8 | 13617 | 13624 | *nad7* (intron) |
|  | (TC)4 | 8 | 14196 | 14203 | IGS (*nad7, rpl2*) |
|  | (AG)5 | 10 | 16271 | 16280 | IGS (*nad7, rpl2*) |
|  | (TC)4 | 8 | 19609 | 19616 | IGS (*nad7, rpl2*) |
|  | (GA)4 | 8 | 20410 | 20417 | IGS (*nad7, rpl2*) |
|  | (GA)4 | 8 | 25922 | 25929 | *rpl2* |
|  | (CT)4 | 8 | 27969 | 27976 | IGS (*rpl2, nad5*) |
|  | (CT)4 | 8 | 28124 | 28131 | IGS (*rpl2, nad5*) |
|  | (CT)4 | 8 | 41122 | 41129 | IGS (*trnV-GAC, cox1*) |
|  | (CT)4 | 8 | 48494 | 48501 | IGS (*trnV-GAC, cox1*) |
|  | (GC)4 | 8 | 52732 | 52739 | IGS (*trnV-GAC, cox1*) |
|  | (CT)4 | 8 | 55682 | 55689 | IGS (*cox1, ccmC*) |
|  | (AC)5 | 10 | 55848 | 55857 | IGS (*cox1, ccmC*) |
|  | (TC)4 | 8 | 59714 | 59721 | IGS (*cox1, ccmC*) |
|  | (AT)4 | 8 | 62202 | 62209 | IGS (*cox1, ccmC*) |
|  | (CT)4 | 8 | 62841 | 62848 | IGS (*cox1, ccmC*) |
|  | (TC)4 | 8 | 63344 | 63351 | IGS (*cox1, ccmC*) |
|  | (TC)4 | 8 | 65657 | 65664 | IGS (*cox1, ccmC*) |
|  | (GA)4 | 8 | 74395 | 74402 | IGS (*ccmC, trnM-CAT*) |
|  | (TG)4 | 8 | 76444 | 76451 | IGS (*ccmC, trnM-CAT*) |
|  | (AG)4 | 8 | 76717 | 76724 | IGS (*ccmC, trnM-CAT*) |
|  | (CT)4 | 8 | 79928 | 79935 | IGS (*ccmC, trnM-CAT*) |
|  | (AT)4 | 8 | 91069 | 91076 | IGS (*trnM-CAT,ccmFc*) |
|  | (GA)4 | 8 | 92683 | 92690 | IGS (*trnM-CAT,ccmFc*) |
|  | (CT)4 | 8 | 93117 | 93124 | IGS (*trnM-CAT,ccmFc*) |
|  | (AG)4 | 8 | 95934 | 95941 | IGS (*trnM-CAT,ccmFc*) |
|  | (TC)4 | 8 | 99298 | 99305 | IGS (*ccmFc,trnS-GCA*) |
|  | (AG)5 | 10 | 107619 | 107628 | IGS (*ccmFc,trnS-GCA*) |
|  | (TG)5 | 10 | 113743 | 113752 | IGS (*ccmFc,trnS-GCA*) |
|  | (TA)4 | 8 | 115677 | 115684 | IGS (*ccmFc,trnS-GCA*) |
|  | (AG)4 | 8 | 116577 | 116584 | IGS (*ccmFc,trnS-GCA*) |
|  | (AG)4 | 8 | 124306 | 124313 | IGS (*trnS-GCA,trnD-GTC*) |
|  | (CT)5 | 10 | 130043 | 130052 | IGS (*trnD-GTC,ccmFn*) |
|  | (AG)4 | 8 | 133148 | 133155 | IGS (*trnD-GTC,ccmFn*) |
|  | (GA)4 | 8 | 141383 | 141390 | IGS (*trnD-GTC,ccmFn*) |
|  | (AG)4 | 8 | 143697 | 143704 | *ccmFn* (intron) |
|  | (AG)4 | 8 | 150344 | 150351 | IGS (*ccmFn,atp8*) |
|  | (TC)4 | 8 | 158283 | 158290 | IGS (*atp8,nad5*) |
|  | (TC)4 | 8 | 161454 | 161461 | IGS (*nad5,trnK-TTT*) |
|  | (TA)4 | 8 | 163094 | 163101 | IGS (*nad5,trnK-TTT*) |
|  | (AG)4 | 8 | 176234 | 176241 | IGS (*trnK-TTT,atp6*) |
|  | (CT)4 | 8 | 176537 | 176544 | IGS (*trnK-TTT,atp6*) |
|  | (GA)4 | 8 | 182939 | 182946 | *nad3* |
|  | (TC)4 | 8 | 189293 | 189300 | IGS (*trnS-TGA,nad4*) |
|  | (CT)5 | 10 | 189466 | 189475 | IGS (*trnS-TGA,nad4*) |
|  | (GT)4 | 8 | 190413 | 190420 | IGS (*trnS-TGA,nad4*) |
|  | (TC)4 | 8 | 198548 | 198555 | *nad4* (intron) |
|  | (AC)4 | 8 | 199188 | 199195 | *nad4* (intron) |
|  | (CT)5 | 10 | 200497 | 200506 | IGS (*nad4,rps7*) |
|  | (CT)5 | 10 | 200719 | 200728 | IGS (*nad4,rps7*) |
|  | (AG)4 | 8 | 205493 | 205500 | IGS (*rps7,mttB*) |
|  | (TC)4 | 8 | 206408 | 206415 | IGS (*rps7,mttB*) |
|  | (CT)4 | 8 | 208077 | 208084 | IGS (*rps7,mttB*) |
|  | (CT)4 | 8 | 209606 | 209613 | IGS (*rps7,mttB*) |
|  | (TC)4 | 8 | 220806 | 220813 | IGS (*rps7,mttB*) |
|  | (AG)4 | 8 | 224416 | 224423 | IGS (*rps7,mttB*) |
|  | (AT)4 | 8 | 226694 | 226701 | IGS (*rps7,mttB*) |
|  | (CT)5 | 10 | 227682 | 227691 | IGS (*mttB,nad5*) |
|  | (CT)4 | 8 | 233468 | 233475 | IGS (*mttB,nad5*) |
|  | (TC)4 | 8 | 255517 | 255524 | IGS (*trnP-TGG,rpl10*) |
|  | (TA)4 | 8 | 261635 | 261642 | IGS (*trnP-TGG,rpl10*) |
|  | (TA)4 | 8 | 267818 | 267825 | IGS (*nad6,nad1*) |
|  | (GA)4 | 8 | 270080 | 270087 | IGS (*nad6,nad1*) |
|  | (AT)5 | 10 | 274124 | 274133 | IGS (*nad6,nad1*) |
|  | (TC)5 | 10 | 279390 | 279399 | IGS (*nad1,ccmB*) |
|  | (CT)4 | 8 | 281532 | 281539 | IGS (*nad1,ccmB*) |
|  | (TC)4 | 8 | 283492 | 283499 | IGS (*nad1,ccmB*) |
|  | (TG)4 | 8 | 289412 | 289419 | IGS (*ccmB,trnH-GTG*) |
|  | (AC)4 | 8 | 289945 | 289952 | IGS (*ccmB,trnH-GTG*) |
|  | (GA)4 | 8 | 298991 | 298998 | IGS (*ccmB,trnH-GTG*) |
|  | (CG)4 | 8 | 299593 | 299600 | IGS (*ccmB,trnH-GTG*) |
|  | (TC)5 | 10 | 301058 | 301067 | IGS (*ccmB,trnH-GTG*) |
|  | (AG)4 | 8 | 302150 | 302157 | IGS (*ccmB,trnH-GTG*) |
|  | (CT)4 | 8 | 304320 | 304327 | IGS (*ccmB,trnH-GTG*) |
|  | (TC)4 | 8 | 306554 | 306561 | IGS (*ccmB,trnH-GTG*) |
|  | (TA)4 | 8 | 312877 | 312884 | IGS (*trnH-GTG,atp4*) |
|  | (TC)4 | 8 | 313934 | 313941 | IGS (*trnH-GTG,atp4*) |
|  | (CT)4 | 8 | 318431 | 318438 | IGS (*nad4L,trnC-GCA*) |
|  | (AG)4 | 8 | 318641 | 318648 | IGS (*nad4L,trnC-GCA*) |
|  | (TA)4 | 8 | 320709 | 320716 | IGS (*nad4L,trnC-GCA*) |
|  | (AC)4 | 8 | 322256 | 322263 | IGS (*nad4L,trnC-GCA*) |
|  | (AG)4 | 8 | 329492 | 329499 | IGS (*nad4L,trnC-GCA*) |
|  | (TA)4 | 8 | 330364 | 330371 | IGS (*nad4L,trnC-GCA*) |
|  | (TA)4 | 8 | 333612 | 333619 | IGS (*trnN-GTT,trnY-GTA)* |
|  | (CG)4 | 8 | 334198 | 334205 | *nad2* (intron) |
|  | (CT)4 | 8 | 343287 | 343294 | *nad2* (intron) |
|  | (TA)4 | 8 | 344983 | 344990 | *nad2* (intron) |
|  | (AG)4 | 8 | 345417 | 345424 | *nad2* (intron) |
|  | (CT)4 | 8 | 353620 | 353627 | IGS (*nad2,trnM-CAT*) |
|  | (CT)5 | 10 | 354912 | 354921 | IGS (*nad2,trnM-CAT*) |
|  | (TC)6 | 12 | 360909 | 360920 | IGS (*nad2,trnM-CAT*) |
|  | (GA)4 | 8 | 362073 | 362080 | IGS (*nad2,trnM-CAT*) |
|  | (AG)5 | 10 | 362232 | 362241 | IGS (*nad2,trnM-CAT*) |
|  | (AG)4 | 8 | 362935 | 362942 | IGS (*nad2,trnM-CAT*) |
|  | (TC)5 | 10 | 363731 | 363740 | IGS (*nad2,trnM-CAT*) |
|  | (GA)4 | 8 | 366949 | 366956 | IGS (*nad2,trnM-CAT*) |
|  | (AG)4 | 8 | 367388 | 367395 | IGS (*nad2,trnM-CAT*) |
|  | (AG)4 | 8 | 369998 | 370005 | IGS (*nad2,trnM-CAT*) |
|  | (AG)4 | 8 | 379936 | 379943 | IGS (*nad2,trnM-CAT*) |
|  | (TC)4 | 8 | 384253 | 384260 | IGS (*trnM-CAT,cox3*) |
|  | (TC)4 | 8 | 388544 | 388551 | IGS (*trnM-CAT,cox3*) |
|  | (AG)4 | 8 | 389266 | 389273 | IGS (*trnM-CAT,cox3*) |
|  | (TC)4 | 8 | 398541 | 398548 | IGS (*sdh4,rrn5*) |
|  | (AG)4 | 8 | 407517 | 407524 | IGS (*rrnS,rpl16*) |
|  | (CT)4 | 8 | 407896 | 407903 | IGS (*rrnS,rpl16*) |
|  | (CT)4 | 8 | 408088 | 408095 | IGS (*rrnS,rpl16*) |
|  | (TC)4 | 8 | 417970 | 417977 | IGS (*rrnS,rpl16*) |
|  | (GA)6 | 12 | 421515 | 421526 | IGS (*rpl16,trnP-TGG*) |
|  | (AG)4 | 8 | 421748 | 421755 | IGS (*rpl16,trnP-TGG*) |
|  | (TG)4 | 8 | 427014 | 427021 | IGS (*rpl16,trnP-TGG*) |
|  | (TC)4 | 8 | 430107 | 430114 | IGS (*rpl16,trnP-TGG*) |
|  | (CT)4 | 8 | 433337 | 433344 | IGS (*rpl16,trnP-TGG*) |
|  | (TC)4 | 8 | 434899 | 434906 | IGS (*rpl16,trnP-TGG*) |
|  | (AT)4 | 8 | 438714 | 438721 | IGS (*trnS-GCT,nad9*) |
|  | (GT)4 | 8 | 440592 | 440599 | IGS (*trnS-GCT,nad9*) |
|  | (CT)5 | 10 | 440722 | 440731 | IGS (*trnS-GCT,nad9*) |
|  | (CT)4 | 8 | 447451 | 447458 | IGS (*trnW-CCA,trnE-TTC*) |
|  | (AG)6 | 12 | 449274 | 449285 | IGS (*trnW-CCA,trnE-TTC*) |
|  | (GA)4 | 8 | 449667 | 449674 | IGS (*trnW-CCA,trnE-TTC*) |
|  | (AG)4 | 8 | 450663 | 450670 | IGS (*trnW-CCA,trnE-TTC*) |
|  | (AT)4 | 8 | 450877 | 450884 | IGS (*trnW-CCA,trnE-TTC*) |
|  | (AT)4 | 8 | 466230 | 466237 | IGS (*trnQ-TTG,trnG-GCC*) |
|  | (TC)4 | 8 | 467043 | 467050 | IGS (*trnQ-TTG,trnG-GCC*) |
|  | (GA)4 | 8 | 469155 | 469162 | IGS (*trnG-GCC,cox2*) |
|  | (AG)4 | 8 | 469471 | 469478 | IGS (*trnG-GCC,cox2*) |
|  | (CT)4 | 8 | 471731 | 471738 | IGS (*trnG-GCC,cox2*) |
|  | (CG)4 | 8 | 473339 | 473346 | IGS (*trnG-GCC,cox2*) |
|  | (GA)4 | 8 | 479604 | 479611 | IGS (*trnG-GCC,cox2*) |
|  | (TC)4 | 8 | 480163 | 480170 | IGS (*trnG-GCC,cox2*) |
|  | (AG)4 | 8 | 483579 | 483586 | IGS (*cox2,trnM-CAT*) |
|  | (AG)4 | 8 | 484327 | 484334 | IGS (*cox2,trnM-CAT*) |
|  | (TC)4 | 8 | 484644 | 484651 | IGS (*cox2,trnM-CAT*) |
|  | (AG)4 | 8 | 492767 | 492774 | IGS (*cox2,trnM-CAT*) |
|  | (TC)4 | 8 | 498620 | 498627 | IGS (*cox2,trnM-CAT*) |
|  | (AT)4 | 8 | 507754 | 507761 | IGS (*trnM-CAT,cob*) |
|  | (TC)4 | 8 | 511188 | 511195 | IGS (*cob,nad1*) |
|  | (GA)4 | 8 | 516652 | 516659 | IGS (*cob,nad1*) |
|  | (TC)4 | 8 | 518846 | 518853 | IGS (*cob,nad1*) |
|  | (TC)4 | 8 | 524505 | 524512 | IGS (*cob,nad1*) |
|  | (TA)4 | 8 | 530196 | 530203 | *nad1* |
|  | (AG)4 | 8 | 530640 | 530647 | IGS (*nad1,rrnL*) |
|  | (AG)4 | 8 | 541038 | 541045 | IGS (*atp1,atp9*) |
|  | (AG)4 | 8 | 543409 | 543416 | IGS (*atp1,atp9*) |
|  | (AG)4 | 8 | 545292 | 545299 | IGS (*atp1,atp9*) |
|  | (AG)4 | 8 | 549525 | 549532 | IGS (*atp1,atp9*) |
|  | (GA)4 | 8 | 551411 | 551418 | IGS (*atp1,atp9*) |
|  | (AG)4 | 8 | 559956 | 559963 | IGS (*atp1,atp9*) |
|  | (TC)4 | 8 | 562509 | 562516 | IGS (*atp1,atp9*) |
|  | (AG)4 | 8 | 563118 | 563125 | IGS (*atp1,atp9*) |
|  | (AT)4 | 8 | 569076 | 569083 | IGS (*atp1,atp9*) |
|  | (AG)5 | 10 | 579755 | 579764 | IGS (*atp9,rps3*) |
|  | (AT)4 | 8 | 587347 | 587354 | IGS (*atp9,rps3*) |
|  | (AG)6 | 12 | 589567 | 589578 | IGS (*atp9,rps3*) |
|  | (TA)4 | 8 | 596511 | 596518 | IGS (*rps3, nad7*) |
| **trimer** | (AAG)4 | 12 | 25208 | 25219 | IGS (*nad7, rpl2*) |
|  | (AGC)4 | 12 | 37094 | 37105 | IGS (*trnV-GAC, cox1*) |
|  | (ACT)4 | 12 | 75918 | 75929 | IGS (*ccmC, trnM-CAT*) |
|  | (TCT)4 | 12 | 91439 | 91450 | IGS (*trnM-CAT,ccmFc*) |
|  | (AAG)4 | 12 | 166790 | 166801 | IGS (*nad5,trnK-TTT*) |
|  | (AGT)4 | 12 | 264619 | 264630 | IGS (*nad6,nad1*) |
|  | (CTA)4 | 12 | 282818 | 282829 | IGS (*nad1,ccmB*) |
|  | (GAA)4 | 12 | 311136 | 311147 | IGS (*trnH-GTG,atp4*) |
|  | (TTC)4 | 12 | 357618 | 357629 | IGS (*nad2,trnM-CAT*) |
|  | (AAG)4 | 12 | 377812 | 377823 | IGS (*nad2,trnM-CAT*) |
|  | (AGC)4 | 12 | 429436 | 429447 | IGS (*rpl16,trnP-TGG*) |
|  | (AAT)4 | 12 | 474743 | 474754 | IGS (*trnG-GCC,cox2*) |
|  | (AGT)4 | 12 | 559036 | 559047 | IGS (*atp1,atp9*) |
|  | (GAA)4 | 12 | 572166 | 572177 | IGS (*atp1,atp9*) |
|  | (TAT)4 | 12 | 588334 | 588345 | IGS (*atp9,rps3*) |
|  | (GAC)4 | 12 | 591146 | 591157 | *rps3* |
|  | (TTC)4 | 12 | 595263 | 595274 | IGS (*rps3, nad7*) |
| **tetramer** | (CTTT)3 | 12 | 18092 | 18103 | IGS (*nad7, rpl2*) |
|  | (AAGA)3 | 12 | 22663 | 22674 | IGS (*nad7, rpl2*) |
|  | (AAAG)3 | 12 | 30116 | 30127 | *nad5* (intron) |
|  | (CTGT)3 | 12 | 49554 | 49565 | IGS (*trnV-GAC, cox1*) |
|  | (TCTG)3 | 12 | 50646 | 50657 | IGS (*trnV-GAC, cox1*) |
|  | (ATGG)3 | 12 | 60450 | 60461 | IGS (*cox1, ccmC*) |
|  | (CTTT)3 | 12 | 76941 | 76952 | IGS (*ccmC, trnM-CAT*) |
|  | (CCTT)3 | 12 | 99110 | 99121 | IGS (*ccmFc,trnS-GCA*) |
|  | (ATAA)3 | 12 | 107793 | 107804 | IGS (*ccmFc,trnS-GCA*) |
|  | (CAAT)3 | 12 | 109566 | 109577 | IGS (*ccmFc,trnS-GCA*) |
|  | (CTTA)3 | 12 | 159611 | 159622 | IGS (*atp8,nad5*) |
|  | (TGCT)3 | 12 | 160527 | 160538 | IGS (*nad5,trnK-TTT*) |
|  | (CCTA)3 | 12 | 168782 | 168793 | IGS (*nad5,trnK-TTT*) |
|  | (ATAA)3 | 12 | 181303 | 181314 | IGS (*trnK-TTT,atp6*) |
|  | (GCTA)3 | 12 | 183454 | 183465 | IGS (*nad3,nad1*) |
|  | (GGCG)3 | 12 | 195594 | 195605 | *nad4* (intron) |
|  | (AGAA)3 | 12 | 208475 | 208486 | IGS (*rps7,mttB*) |
|  | (GCCT)3 | 12 | 208655 | 208666 | IGS (*rps7,mttB*) |
|  | (AGAA)3 | 12 | 209222 | 209233 | IGS (*rps7,mttB*) |
|  | (GCTT)3 | 12 | 210097 | 210108 | IGS (*rps7,mttB*) |
|  | (TACC)3 | 12 | 219738 | 219749 | IGS (*rps7,mttB*) |
|  | (CGGC)3 | 12 | 238614 | 238625 | IGS (*mttB,nad5*) |
|  | (ACTA)3 | 12 | 259428 | 259439 | IGS (*trnP-TGG,rpl10*) |
|  | (AAGA)3 | 12 | 266144 | 266155 | IGS (*nad6,nad1*) |
|  | (ATTA)3 | 12 | 289813 | 289824 | IGS (*atp1,trnH-GTG*) |
|  | (CTTT)6 | 24 | 296291 | 296314 | IGS (*atp1,trnH-GTG*) |
|  | (TTTC)3 | 12 | 301670 | 301681 | IGS (*atp1,trnH-GTG*) |
|  | (TTAA)3 | 12 | 309718 | 309729 | IGS (*trnH-GTG,atp4*) |
|  | (GAAA)3 | 12 | 317075 | 317086 | IGS (*atp4,nad4L*) |
|  | (TTCA)3 | 12 | 332951 | 332962 | IGS (*trnN-GTT,trnY-GTA)* |
|  | (GAAT)3 | 12 | 352842 | 352853 | IGS (*nad2,trnM-CAT*) |
|  | (TTCT)3 | 12 | 355207 | 355218 | IGS (*nad2,trnM-CAT*) |
|  | (AGAA)3 | 12 | 365308 | 365319 | IGS (*nad2,trnM-CAT*) |
|  | (TGAC)3 | 12 | 374349 | 374360 | IGS (*nad2,trnM-CAT*) |
|  | (CCTT)3 | 12 | 374809 | 374820 | IGS (*nad2,trnM-CAT*) |
|  | (TCTT)3 | 12 | 375924 | 375935 | IGS (*nad2,trnM-CAT*) |
|  | (CTTT)3 | 12 | 383668 | 383679 | IGS (*trnM-CAT,cox3*) |
|  | (AAGA)3 | 12 | 397573 | 397584 | IGS (*sdh4,rrn5*) |
|  | (TTTC)3 | 12 | 401488 | 401499 | *rrnS* |
|  | (GCTT)3 | 12 | 409441 | 409452 | IGS (*rrnS,rpl16*) |
|  | (AAAG)3 | 12 | 418742 | 418753 | IGS (*rrnS,rpl16*) |
|  | (AAAG)3 | 12 | 434404 | 434415 | IGS (*rpl16,trnP-TGG*) |
|  | (CCCA)3 | 12 | 463387 | 463398 | IGS (*trnE-TTC,trnQ-TTG*) |
|  | (CTTG)3 | 12 | 511704 | 511715 | IGS (*cob,nad1*) |
|  | (CTTT)3 | 12 | 512419 | 512430 | IGS (*cob,nad1*) |
|  | (TTCT)4 | 16 | 519323 | 519338 | IGS (*cob,nad1*) |
|  | (CTTT)3 | 12 | 548373 | 548384 | IGS (*atp1,atp9*) |
|  | (TTTC)3 | 12 | 552673 | 552684 | IGS (*atp1,atp9*) |
|  | (CTTA)4 | 16 | 557793 | 557808 | IGS (*atp1,atp9*) |
| **pentamer** | (CCTAT)3 | 15 | 27329 | 27343 | *rpl2* (intron) |
|  | (TATGG)3 | 15 | 48021 | 48035 | IGS (*trnV-GAC, cox1*) |
|  | (TTAAA)3 | 15 | 112130 | 112144 | IGS (*ccmFc,trnS-GCA*) |
|  | (CCTAA)3 | 15 | 170981 | 170995 | IGS (*trnK-TTT,atp6*) |
|  | (CTCTT)3 | 15 | 190961 | 190975 | IGS (*trnS-TGA,nad4*) |
|  | (TTTTC)3 | 15 | 191542 | 191556 | IGS (*trnS-TGA,nad4*) |
|  | (GTAAA)4 | 20 | 378965 | 378984 | IGS (*nad2,trnM-CAT*) |
|  | (ATAAG)3 | 15 | 453432 | 453446 | IGS (*trnE-TTC,trnQ-TTG*) |
|  | (CTAGT)3 | 15 | 529212 | 529226 | *nad1* (intron) |
| **hexamer** | (TATGGA)3 | 18 | 288554 | 288571 | IGS (*ccmB,atp1*) |

**Table S2**. Comparison of basic features among 35 mitochondrial genomes.

| Name | Size (bp) | GC% | Protein | rRNA | tRNA | Gene |
| --- | --- | --- | --- | --- | --- | --- |
| *Anomodon rugelii* | 104,239 | 41.20 | 46 | 3 | 24 | 76 |
| *Physcomitrella patens* | 105,340 | 40.57 | 42 | 3 | 24 | 69 |
| *Bucklandiella orthotrichacea* | 107,215 | 39.93 | 39 | 3 | 24 | 66 |
| *Tetraphis pellucida* | 107,730 | 42.53 | 41 | 3 | 24 | 68 |
| *Atrichum angustatum* | 115,146 | 40.51 | 40 | 3 | 24 | 67 |
| *Aneura pinguis* | 165,603 | 47.44 | 40 | 3 | 27 | 70 |
| *Pleurozia purpurea* | 168,526 | 45.37 | 69 | 3 | 28 | 101 |
| *Nothoceros aenigmaticus* | 184,908 | 46.01 | 48 | 3 | 18 | 80 |
| *Phaeoceros laevis* | 209,482 | 44.60 | 38 | 3 | 20 | 73 |
| *Brassica nigra* | 232,407 | 45.31 | 77 | 3 | 19 | 99 |
| *Raphanus sativus* | 258,426 | 45.21 | 35 | 3 | 18 | 56 |
| *Helianthus annuus* | 300,945 | 45.05 | 26 | 3 | 15 | 44 |
| *Ajuga reptans* | 352,069 | 45.10 | 30 | 3 | 17 | 50 |
| *Arabidopsis thaliana* | 366,924 | 44.77 | 117 | 3 | 21 | 131 |
| *Citrullus lanatus* | 379,236 | 45.08 | 39 | 3 | 18 | 63 |
| *Lotus japonicus* | 380,861 | 45.40 | 34 | 3 | 20 | 65 |
| *Beta macrocarpa* | 385,220 | 43.89 | 156 | 5 | 26 | 191 |
| *Glycine max* | 402,558 | 45.03 | 88 | 3 | 19 | 110 |
| *Batis maritima* | 403,930 | 45.41 | 112 | 3 | 20 | 148 |
| *Vigna angularis* | 404,466 | 45.19 | 26 | 3 | 16 | 51 |
| *Phlegmariurus squarrosus* | 413,530 | 44.16 | 147 | 6 | 34 | 193 |
| *Nicotiana tabacum* | 430,597 | 44.96 | 156 | 4 | 23 | 183 |
| *Triticum aestivum* | 452,528 | 44.35 | 39 | 9 | 25 | 78 |
| *Carica papaya* | 476,890 | 45.12 | 39 | 3 | 19 | 61 |
| *Salvia miltiorrhiza* | 499,236 | 44.39 | 138 | 5 | 22 | 167 |
| *Hyoscyamus niger* | 501,401 | 45.18 | 38 | 4 | 28 | 71 |
| *Capsicum annuum* | 511,530 | 44.52 | 193 | 3 | 25 | 221 |
| *Zea luxurians* | 539,368 | 43.93 | 32 | 3 | 17 | 54 |
| *Viscum album* | 565,432 | 45.87 | 12 | 1 | 6 | 19 |
| *Zea mays NB* | 569,630 | 43.93 | 163 | 4 | 29 | 213 |
| *Gossypium barbadense* | 677,434 | 44.98 | 36 | 3 | 24 | 63 |
| *Asclepias syriaca* | 682,498 | 43.43 | 37 | 3 | 27 | 72 |
| *Populus tremula* | 783,442 | 44.75 | 33 | 3 | 22 | 59 |
| *Welwitschia mirabilis* | 978,846 | 53.02 | 29 | 3 | 8 | 40 |
| *Cucurbita pepo* | 982,833 | 42.80 | 38 | 3 | 13 | 55 |

**Table S3**. The list of mitochondrial genome sequences used in phylogenetic study.

| **Taxon** | **family** | **order** | **class** | **phylum** | **RefSeq** |
| --- | --- | --- | --- | --- | --- |
| *Aegilops speltoides* | Poaceae | Poales | Monocots | Angiosperms | NC_022666 |
| *Ajuga reptans* | Lamiaceae | Lamiales | Eudicots | Angiosperms | NC_023103 |
| *Batis maritima* | Bataceae | Brassicales | Eudicots | Angiosperms | NC_024429 |
| *Beta macrocarpa* | Amaranthaceae | Caryophyllales | Eudicots | Angiosperms | NC_015994 |
| *Boea hygrometrica* | Gesneriaceae | Lamiales | Eudicots | Angiosperms | NC_016741 |
| *Carica papaya* | Caricaceae | Brassicales | Eudicots | Angiosperms | NC_012116 |
| *Citrullus lanatus* | Cucurbitaceae | Cucurbitales | Eudicots | Angiosperms | NC_014043 |
| *Cucumis sativus* | Cucurbitaceae | Cucurbitales | Eudicots | Angiosperms | NC_016005 |
| *Cucurbita pepo* | Cucurbitaceae | Cucurbitales | Eudicots | Angiosperms | NC_014050 |
| *Ginkgo biloba* | Ginkgoaceae | Ginkgoales | Eudicots | Gymnosperms | NC_027976 |
| *Gossypium barbadense* | Malvaceae | Malvales | Eudicots | Angiosperms | NC_028254 |
| *Hyoscyamus niger* | Solanaceae | Solanales | Eudicots | Angiosperms | NC_026515 |
| *Liriodendron tulipifera* | Magnoliaceae | Magnoliales | Eudicots | Angiosperms | NC_021152 |
| *Phoenix dactylifera* | Arecaceae | Arecales | Monocots | Angiosperms | NC_016740 |
| *Populus tremula* | Salicaceae | Malpighiales | Eudicots | Angiosperms | NC_028096 |
| *Salvia miltiorrhiza* | Lamiaceae | Lamiales | Eudicots | Angiosperms | NC_023209 |
| *Silene latifolia* | Caryophyllaceae | Caryophyllales | Eudicots | Angiosperms | NC_014487 |
| *Sorghum bicolor* | Poaceae | Poales | Monocots | Angiosperms | NC_008360 |
| *Vitis vinifera* | Vitaceae | Vitales | Eudicots | Angiosperms | NC_012119 |
| *Zea luxurians* | Poaceae | Poales | Monocots | Angiosperms | NC_008333 |
| *Zea mays subsp parviglumis* | Poaceae | Poales | Monocots | Angiosperms | NC_008332 |
| *Zea perennis* | Poaceae | Poales | Monocots | Angiosperms | NC_008331 |
